# Proteomic Analysis of Anti-Cancerous Scopularide Production by a Marine *Microascus brevicaulis* Strain and Its UV Mutant

**DOI:** 10.1371/journal.pone.0140047

**Published:** 2015-10-13

**Authors:** Annemarie Kramer, Hans Christian Beck, Abhishek Kumar, Lars Peter Kristensen, Johannes F. Imhoff, Antje Labes

**Affiliations:** 1 Research Unit Marine Ecology, GEOMAR Helmholtz Centre for Ocean Research Kiel, Kiel, Germany; 2 Centre for Clinical Proteomics, Department for Clinical Biochemistry and Pharmacology, Odense University Hospital, Odense, Denmark; 3 Department for Botany and Molecular Biology, Institute of Botany, Christian-Albrechts University of Kiel, Kiel, Germany; University Paris South, FRANCE

## Abstract

The marine fungus *Microascus brevicaulis* strain LF580 is a non-model secondary metabolite producer with high yields of the two secondary metabolites scopularides A and B, which exhibit distinct activities against tumour cell lines. A mutant strain was obtained using UV mutagenesis, showing faster growth and differences in pellet formation besides higher production levels. Here, we show the first proteome study of a marine fungus. Comparative proteomics were applied to gain deeper understanding of the regulation of production and of the physiology of the wild type strain and its mutant. For this purpose, an optimised protein extraction protocol was established. In total, 4759 proteins were identified. The central metabolic pathway of strain LF580 was mapped using the KEGG pathway analysis and GO annotation. Employing iTRAQ labelling, 318 proteins were shown to be significantly regulated in the mutant strain: 189 were down- and 129 upregulated. Proteomics are a powerful tool for the understanding of regulatory aspects: The differences on proteome level could be attributed to limited nutrient availability in the wild type strain due to a strong pellet formation. This information can be applied for optimisation on strain and process level. The linkage between nutrient limitation and pellet formation in the non-model fungus *M*. *brevicaulis* is in consensus with the knowledge on model organisms like *Aspergillus niger* and *Penicillium chrysogenum*.

## Introduction

New secondary metabolites produced by fungi hold a great potential in application for human use [1, 2]. The two cyclodepsipeptides scopularides A and B are produced by an ascomycete, isolated from the inner tissue of the marine sponge *Tethya aurantium* [3]. The fungal strain was originally described as *Scopulariopsis brevicaulis* LF580. Recent studies revealed *S*. *brevicaulis* to be the teleomorph of *Microascus brevicaulis* [4–6]. According to the rules of nomenclature for fungi (6), the name *Microascus brevicaulis* strain LF580 is used within this manuscript.

Both scopularides demonstrated specific activity against the pancreatic tumour cell lines Colo357 and Panc89 as well as the colon tumour cell line HT29 [3]. Both scopularides consist of five amino acids cyclised via a side chain (scopularide A: (*cyclo*-(3-hydroxy-4-methyldecanoyl- Gly–l-Val–d-Leu–l-Ala–l-Phe); scopularide B: *cyclo*-(3-hydroxy-4-methylloctanoyl- Gly–l-Val–d-Leu–l-Ala–l-Phe)). Within the genome analysis of *M*. *brevicaulis* LF580, 17 NRPS genes were identified [7]. The responsible gene cluster for scopularide A includes a non-ribosomal peptide synthetase (NRPS1), a polyketide synthase (PKS2), a CoA ligase, an acyltransferase and a transcription factor [7, 8]. Due to the structural analogy, a synthesis of both scopularides by the same enzyme complex is assumed.

The wild type strain LF580 represents a non-model secondary metabolite producer, exhibiting high yields of the target metabolites (approximately 200 mg L^-1^ for scopularide A [9]). Using an optimised screening approach [10], a UV mutant strain (*M*. *brevicaulis* strain LF580-M26) was detected, which showed an increased production level of the two scopularides under standard conditions, faster and different growth and changes in pellet formation. Thus, this mutant strain LF580-M26 was chosen for a detailed comparison with the wild type strain on proteome level.

Comparative proteome profiling helps to understand metabolic changes as mediation of cellular metabolic activities occurs in principle on protein level [11, 12]. Quantitative proteomics can link physiological changes, growth characteristics and enhanced secondary metabolite production with molecular changes at the proteome level. A quantitative comparison provides novel information on the regulatory pathways involved and contributes to precise strain and process development [13–16]. Despite their prevalence in the biotechnological industry and their significance as pathogens, filamentous fungi received less attention during the initiation and implementation of innovative biological methods and in systems biology in the past [17]. Like in the genomic era, filamentous fungi are stragglers in the proteomic era: In 2008, only a few fungal proteome studies were available, mainly describing classical model species of the two genera *Aspergillus* and *Penicillium* [15]. Today, 250 fungal proteomes are available, representing less than 5% when compared to the available bacterial proteomes (http://www.uniprot.org, August 2015). Molecular whole cell analyses disclose comprehensive insights especially into non-model organisms, providing information of the reaction of the organism to environmental changes. Some studies already gave a glimpse of the huge potential of proteomics: *E*.*g*. the proteome analysis of developmental stages in *Cordyceps militaris* led to concrete starting points for strain development [18], proteome studies of *A*. *fumigatus* shed new light on pathogenicity mechanisms [19], and the comparative proteome analysis of an *A*. *nidulans* strain and its mutant strain revealed the multiple effects of gene deletions [20, 21].

Proteome techniques are rapidly evolving, but many protocols were developed for only a small number of organisms. Nearly all new studies needed an adaptation of the protein extraction methodology. In general, fungi are known to have rigid and complex cell walls, which is why a special focus on cell wall disruption is required during sample preparation [17, 22, 23].

The aim of this study was the proteome based characterisation of the differences between the UV mutant LF580-M26 and its wild type (WT) strain in order to identify the underlying molecular mechanisms for the increased scopularide production by the UV mutant strain. This study is the first proteome analysis of a marine filamentous fungus.

## Results

### Identification of equal sampling points by determination of growth characteristics and scopularide production

**Fig 1:**
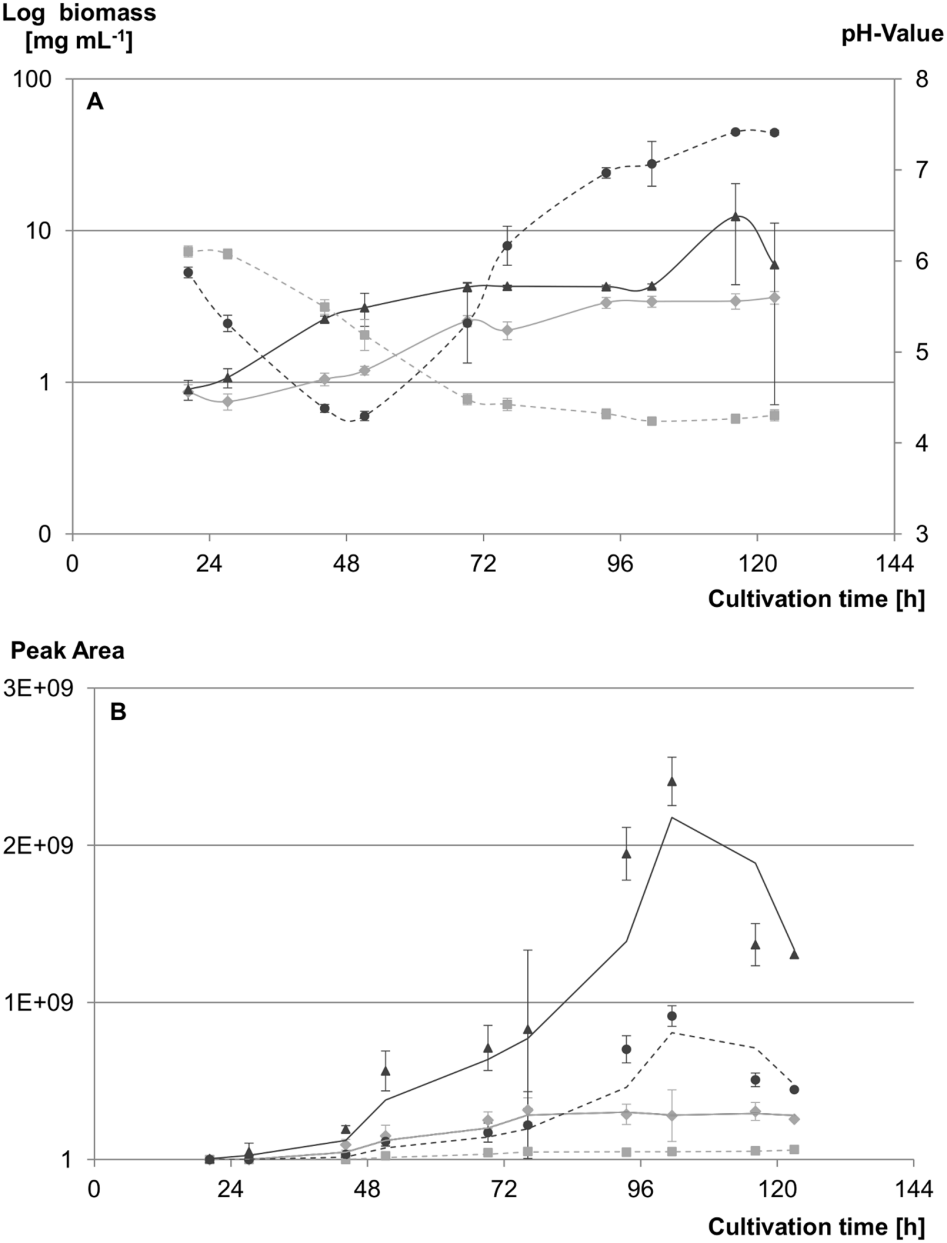
Comparative growth curves of LF580 and LF580-M26. (A) Time series of the biomass (LF580: —◆— (light grey); LF580-M26: —▲— (dark grey)) and pH value (LF580: --■-- (light grey); LF580-M26: --●-- (dark grey)) for the wild type strain LF580 and its mutant strain LF580-M26 are shown. Determinations were performed in triplicates for 6 days. (B) Production levels of scopularide A (LF580: —◆— (light grey); LF580-M26: —▲— (dark grey)) and B (LF580: --■-- (light grey); LF580-M26: --●-- (dark grey)) during the time series are compared on the basis of the peak area of the respective mass signal after analytical HPLC-MS. The curves represent the trend line.

As all changes of cellular metabolic activities do result in changes on protein level, a comparative proteome analysis of strains with different growth behaviour and physiology can only be obtained by synchronising the metabolic trait of the two strains. A comparison of their growth behaviour was used to select the harvest point, where both strains were assumed to be in the same growth and production stage. Growth curves of both strains were obtained in triplicates and showed differences in the growth behaviour of the WT strain LF580 and the mutant strain LF580-M26 (Fig 1A). Obvious differences were velocity, pH, biomass and scopularides production (Fig 1B) in the different growth phases.

The rapid growth phase of the mutant strain LF580-M26 started 20 hours after inoculation. In contrast, the lag phase of the WT strain ended approximately eight hours later. Taking biomass production and pH as markers for fungal growth, the curves differed in duration of the rapid growth phase, being of approximately 24 h for the mutant and 42 h for the WT. These differences led to growth rates of 0.0715 g biomass L^-1^ h^-1^ and 0.0426 g biomass L^-1^ h^-1^ (based on biomass: μX = dX (dt)^-1^) for the mutant strain LF580-M26 and the WT strain, respectively. The biomass production of both strains during that phase showed quite similar values of 1.71 g biomass L^-1^ (mutant strain LF580-M26) and 1.79 g biomass L^-1^ (WT strain). The final yield of both strains was in the same range (LF580: 3.4 g biomass L^-1^, LF580-M26: 4.3 g biomass L^-1^). Similar to the biomass curve, the progression of the pH curve of the mutant strain LF580-M26 was rather exponential in relation to the linear progression of the pH curve of the WT strain. Comparing the point of time when both strains reached the lowest pH level (indicating the end of rapid growth), a difference of approximately 24 h between both strains was observed. The pH value for the WT strain stayed at that level until the end of the experiment (120 h). In contrast, the pH value of the mutant strain LF580-M26 started to increase immediately after reaching the lowest pH level with the same progression it had been falling previously. The pH value reached a maximum of 7.5 at the end of the experiment.

Both strains showed continuous production of the scopularides with similar rates during the rapid growth phase. A drastic change leading to clear differences between the WT and the mutant strain were observed during the transition of the late rapid to the early stationary phase (Fig 1B): The mutant strain LF580-M26 passed through this transition within 7 h, while the WT strain needed approximately 32 h. The scopularide production rate was 50 times higher in the mutant strain during the transition phase. In the stationary growth phase, the factor decreased to approximately 10. In this phase (*i*.*e*. 52 h and 72 h of cultivation for LF580-M26 and LF580, respectively) the production of both scopularides stagnated in the WT strain, whereas the production in the mutant strain still increased until approximately 100 h of cultivation, followed by a decrease of concentration of both scopularides. The characteristic compact pellet formation of the WT strain was not observed for the mutant strain LF580-M26. Pellet formation of the WT strain started at day two after inoculation; at day three approximately 90% of the pellets reached a size of 2–3 mm in diameter; at day four the pellet formation ended up with diameters of 3–4 mm. No significant increase of pellet number could be observed after four days. In contrast, LF580-M26 showed an increasing turbidity of the culture, with filamentous growth turning into the development of hairy pellets (diameter ca. 1 mm). The viscosity of the cultures increased until day five of cultivation.

Considering the observed differences, pH, biomass and scopularides production were used as markers to determine equivalent sampling points: Accordingly, cells were harvested after 68 h (WT strain LF580) and 44 h (mutant strain LF580-M26). At these times, the strains were assumed to be in a comparable growth state, as the pH (LF580: 4.62 ± 0.13; LF580-M26: 4.95 ± 0.17) and biomass (LF580: 2.5 ± 0.2 mg mL^-1^; LF580-M26: 2.6 ± 0.1 mg mL^-1^) values were in the same range.

### Quantitative Protein Extraction as a Prerequisite for Proteome Studies

**Fig 2:**
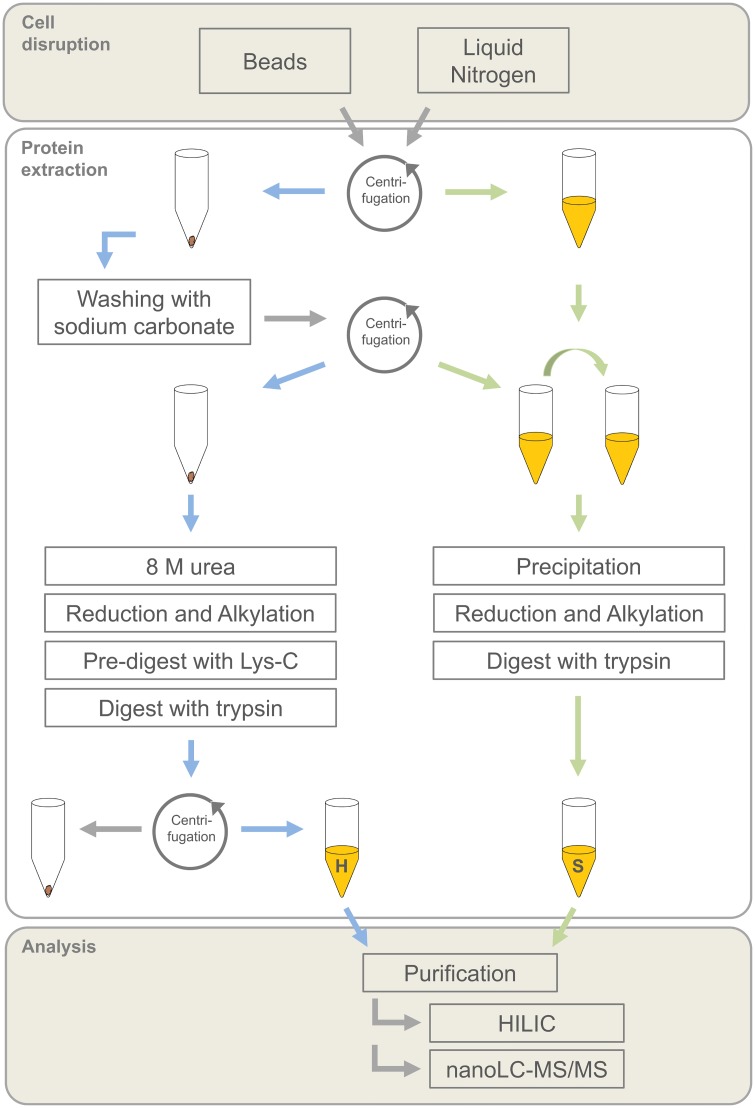
Overview of protein extraction for proteome analysis. Two different cell disruption methods were compared with respect to the number of protein groups identified using nano-LC-MS/MS. An additional focus was set on the solubilisation of the hardly soluble protein fraction (H) beside the soluble protein fraction (S).

In the case of the marine non-model fungus *M*. *brevicaulis* strain LF580 of this study, additional methodological approaches and the validation of protocols were essential to obtain high quality proteome data. A simple standard extraction procedure based on cell disruption by sonication did not lead to a similar quality (pattern) and quantity (intensity) of proteins for the WT and the mutant strain, indicating incomplete extraction. Therefore, an adapted protein extraction protocol was developed and validated. The focus was set on cell disruption and dissolving of the proteins (Fig 2). Two cell disruption procedures were compared: grinding in liquid nitrogen and application of beads. Additionally, the focus was set on the dissolving of the insoluble protein fraction, which pelleted during initial separation.

Applying this adapted protocol, a comparable extraction grade for WT and mutant strain was proven by SDS-PAGE before digest of the final protein extract (results not shown). In order to compare the extraction grades of the different sample preparations (Fig 2), proteome data were analysed sample by sample using nano-LC-MS/MS. Previous studies showed that the entire genome of *M*. *brevicaulis* has a size of approximately 32 Mb, containing 16.298 genes. 1.33% of the genome represented repeats [7, 24]. Here, 4759 proteins were identified (FDR < 0.01 and 1 ≥ peptides per protein) using the developed extraction procedures in both strains. This reflects coverage of approximately 30% of the theoretical protein number. Proteins obtained by liquid nitrogen extraction covered 92.2% of these 4759 proteins. Compared to coverage of 88.1% using bead-based extraction, liquid nitrogen extraction therefore led to an increase of 4.1% of proteins covered. An approximately three times higher increase (11.4%) was achieved by solubilisation of the “hardly soluble” proteins (92.2%), instead of focusing only on the “easily soluble” protein fraction (80.8%). This increase was independent from the original cell disruption method.

### Comparison of the proteomes of wild type strain LF580 and its UV mutant strain LF580-M26

#### Analysis of pathways and protein functions

**Fig 3:**
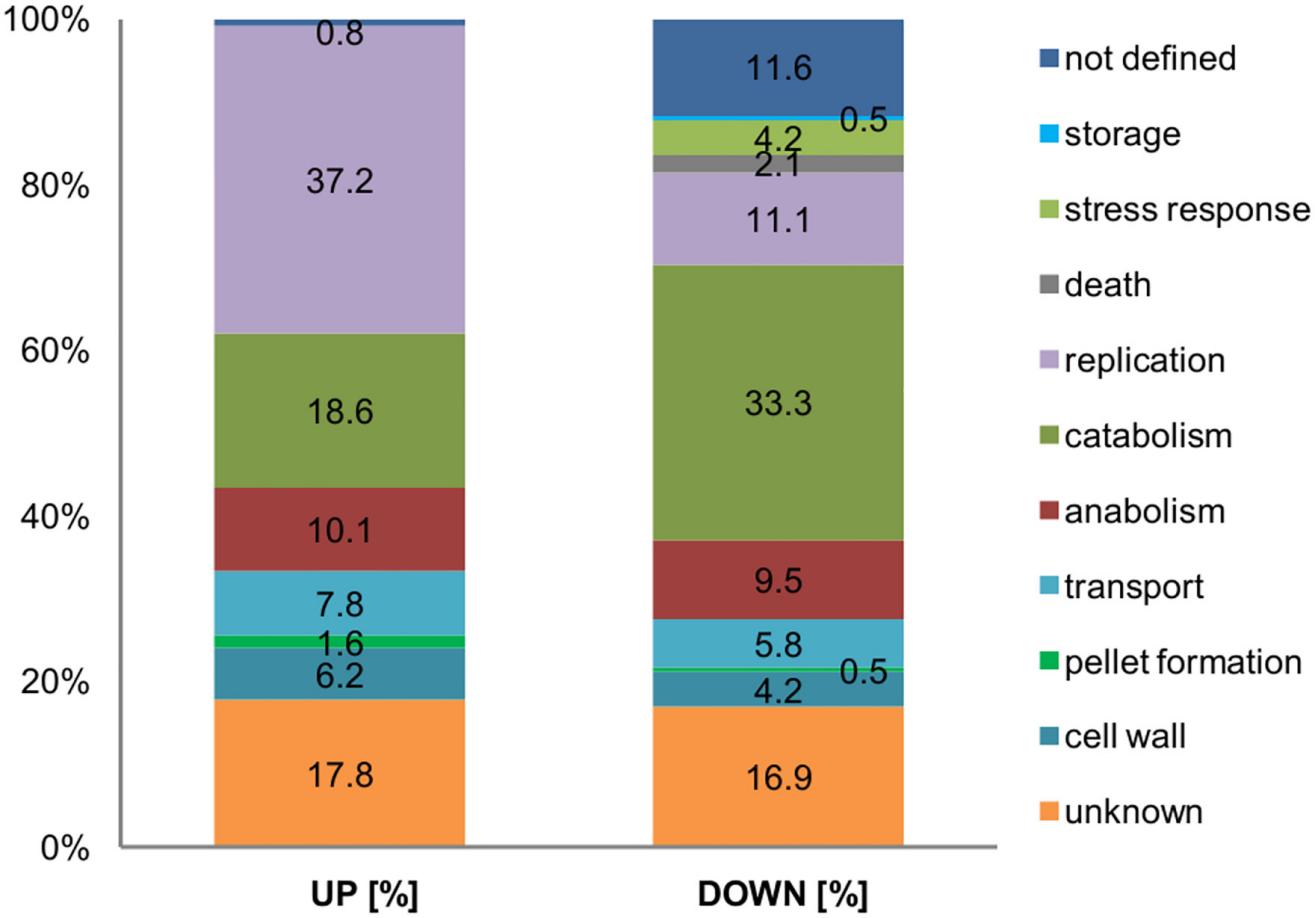
Overview of GO data of mutant strain LF580-M26 in comparison to wild type strain LF580. 3526 proteins were quantified using iTRAQ labelling. 318 (9%) showed a significant regulation in the mutant strain. 129 (3.7%) were upregulated and 189 (5.3%) were downregulated. The bar chart shows the percentage of up- and downregulated proteins sorted to the different categories on the basis of GO annotation.

**Fig 4:**
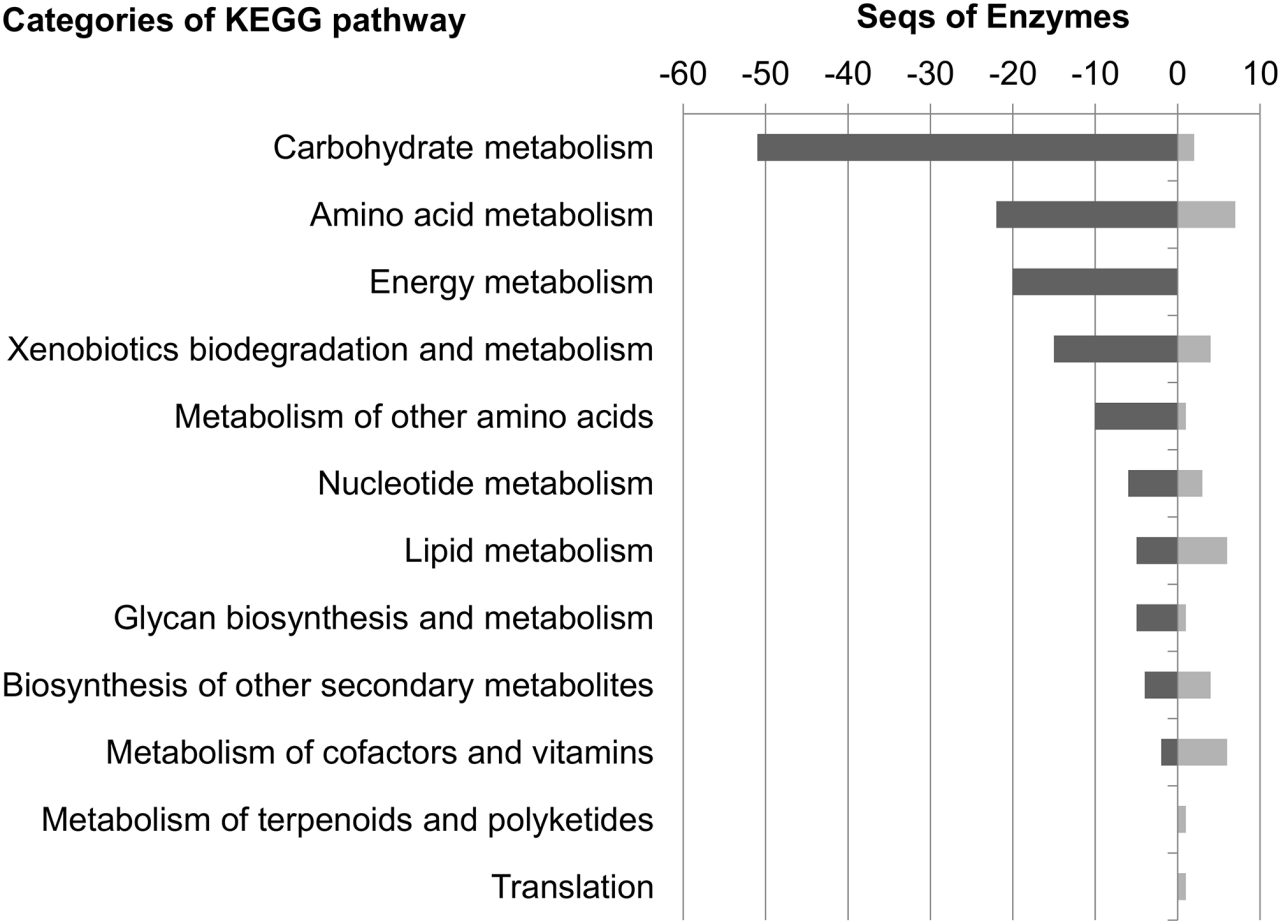
The differentially regulated enzymes were categorised species-independent using the KEGG pathway analyses. Dark grey charts represent the downregulated proteins in the mutant strain, light grey, in contrast, the upregulated ones. The numbers reflect the sequences of enzymes sorted to the different categories.

A combination of all fractions from the liquid nitrogen treated cells was used for quantitative comparison using iTRAQ labelling. 3526 mutual proteins were confidently identified and quantified in the WT and the mutant strain LF580-M26 (FDR < 0.01 and 2 ≥ peptides per protein). 318 proteins were differentially regulated: 129 proteins were upregulated and 189 were downregulated in the mutant strain as compared to the WT strain. For the identification of molecular processes and cellular activities, the set of differentially regulated proteins was subjected to GO analysis [25] (Fig 3). The enzyme functionalities were revealed using KEGG [26], predicting effects of the mutation on cell and metabolic levels (Fig 4).

The evaluation of the GO data was performed by using a set of extended categories, *i*.*e*. sublevels of the GO categories [27]. Main differences were found in the categories of catabolism, pellet formation and replication. The latter two categories showed a distinct upregulation in the mutant with factors of 3.2 (pellet formation) and 3.5 (replication), respectively. However, pellet formation represents a category with a small data set of proteins (1.6% up- and 0.5% downregulated). A clear downregulation was seen for protein groups related to the categories catabolism, storage, stress response and death. 18.6% of the proteins related to the category catabolism were upregulated and 33.3% downregulated. In the category anabolism, approximately the same number of proteins was up- or downregulated: 10.1% and 9.5%, respectively. Slight differences could be seen for the categories cell wall (ratio of 1.5) and transport (ratio of 1.3). Within the categories storage (0.5%), stress response (4.2%) and death (2.1%) only downregulated proteins were found. The number of unknown proteins was almost the same for the up- and downregulated data set (17.8% and 16.9%, respectively). 12.4% (0.8% upregulated, 11.6% downregulated) of the regulated proteins could not be assigned to one of the categories.

KEGG pathway analysis enabled qualification of all necessary enzymes of the central metabolism (see sections below). In general, the expression levels of most enzymes were downregulated in the mutant strain (Fig 4). Within the category “biosynthesis of secondary metabolites” an equal number of enzymes was up- and downregulated. In the categories lipid metabolism, metabolism of cofactors and vitamins, metabolism of terpenoids/polyketides and translation, a larger number of enzymes revealed higher expression levels in the mutant strain.

KEGG and GO data were used for a detailed comparison of the WT strain and the mutant strain LF580-M26.

#### Catabolism

The GO category of catabolism showed a higher number of proteins to be downregulated in the mutant strain (Fig 3). However, downregulation does not reflect a general slow-down of the catabolism. For example, peptidase inhibitor i9 (g7795), an inhibitor for catabolic processes, showed a downregulation in the mutant strain, indicating an enhancement of catabolic processes. The evaluation of the KEGG data confirmed this assumption, because a downregulation was mainly seen on enzyme level for additional pathways and isoenzymes in the mutant strain, probably leading to higher fluxes in the central pathways.

All enzymes of a classical Embden-Meyerhof pathway (glycolysis) were present and active, enabling growth on glucose as expected for a typical eukaryote. Additionally, all enzymes necessary for the initial degradation of other mono- and disaccharides could be qualified. The growth medium contained glucose as carbohydrate source, but soy peptone and yeast extract were sources for additional monomeric sugars (according to suppliers’ certificates of analyses). The expression of all glycolytic enzymes was the same in the WT strain and the mutant strain LF580-M26.

The pentose phosphate pathway (PPP) could be qualified. Most enzymes were downregulated in the mutant strain, such as fructose-biphosphatase (EC 3.1.3.11). The WT strain used both the fructose-biphosphatase (EC 3.1.3.11) and the 6-phosphofructokinase (EC 2.7.1.11). With regard to the oxidative part, the pathway from d-gluconate-6-phosphate to d-ribose-1-phosphate was downregulated in the mutant strain. The WT may benefit from a running PPP, as it generates additional NADPH, which is needed for ammonium uptake as well as for protein biosynthesis. Generally, higher demand for NADPH increases the flux through PPP [28]. The higher level of protein expression in the PPP in the WT indicated such an increase in flux [29].

As expected for a eukaryotic organism, pyruvate generated in glycolysis was channelled via the classical pyruvate dehydrogenase complex into the tricarbonic acid cycle (TCA). The full TCA was operative in the catabolic direction in both strains. However, the mutant strain LF580-M26 showed downregulation of some enzymes: The initial step of the condensation of oxaloacetate and acetyl-CoA to citrate is catalysed by a citrate synthase (EC 2.3.3.8) in the mutant strain. *M*. *brevicaulis* strain LF580-M26 uses the one step isocitrate dehydrogenase isoform (EC 1.1.1.41) generating NADH. The WT additionally expressed the two step isocitrate dehydrogenase (EC 1.1.1.42) generating NADPH. Utilising this enzyme, the WT could raise the NADPH level, *e*.*g*. for increased ammonium uptake or higher levels of translation. The same phenomenon was observed for the succinate dehydrogenase, which is present in the WT strain as EC 1.3.99.1 and EC 1.3.5.1. The latter isoform is membrane bound and part of the respiratory electron transport chain. It was found at the same translation level in both strains. The conversion of CoA-activated carbonic acids (acetate, butyrate, acetoacetate) was downregulated at the level of acetate CoA-transferase (EC 2.8.3.8), leading to a high level of activated substrates in the TCA and subsequent flux in the mutant strain.

CoA-transferase (EC 2.8.3.8) and related enzymes of β-oxidation were downregulated in the mutant strain indicating lower levels of β-oxidation. Downregulation of propionyl-CoA converting enzymes, such as 2-methylcitrate synthase (EC 2.3.3.5) and the subsequent dehydratase (EC 4.2.1.79), coincided with lower levels of β-oxidation, as propionyl-CoA is formed via the degradation of odd-numbered fatty acids. In contrast, the WT showed higher levels of these enzymes, which could be explained by degradation of storage compounds (*i*.*e*. fatty acids) induced by a carbon limitation within the formed pellets.

Enzymes of the respiratory chain (oxidative phosphorylation) were qualified and showed no difference in the protein expression level between mutant and WT strain.

Amino acid degradation and nitrogen metabolism were qualified, indicating that the NADP-glutamate dehydrogenase (EC 1.4.1.4) was used by both strains to assimilate free ammonium from the medium. In the mutant strain, additional ammonium uptake via the glutamine synthetase-glutamine oxoglutarate aminotransferase (GOGAT) cycle was downregulated at the level of conversion from glutamate to glutamine by the glutamine synthetase (EC 6.3.1.2).

The NAD-specific glutamate dehydrogenase (EC 1.4.1.2, converting glutamate to ketoglutarate and ammonium or in reverse) was downregulated in the mutant strain. Such a downregulation was shown for fungal cells not limited in their nitrogen or carbon sources [30, 31]. Vice versa, the NAD-specific glutamate dehydrogenase would be activated, if a strain is limited in one of these sources. As scopularide production is nitrogen dependent [9], the proteome data suggest a nitrogen limitation of the WT in the used medium. In contrast, all pathways for additional gain of nitrogen were downregulated in the mutant strain, which does not seem to be nitrogen limited.

In general, amino acid degradation is active, enabling the strains to grown on peptone containing medium. Conversion of the amino acids to acetyl-CoA for subsequent respiration was enhanced at the level of the respective acetyl-CoA C-acyltransferases (EC 2.3.1.16). Nevertheless, degradation of amino acids necessary for the production of scopularides was downregulated (see below).

#### Anabolism

The evaluation of the GO data revealed a similar number of proteins clustered into the category of anabolism to be up- and downregulated. The KEGG pathway analysis allowed a more detailed view.

The enzymes of the classical Embden-Meyerhof (EM) pathway in eukaryotes usually serve in two directions: for glycolysis and gluconeogenesis. Hence, the presence of a working EM pathway indicates gluconeogenesis operating via the same pathway. As some reactions of the EM pathway are irreversible, additional reactions are necessary for gluconeogenesis: The eukaryotic glucogenetic key enzymes 1,6-fructose bisphosphatase (EC 3.1.3.11) and phosphoenolpyruvate carboxykinase (EC 4.1.1.49) were qualified and shown to be downregulated in the mutant strain. The third enzyme, a glucose-6 phosphatase (EC 3.1.3.9), could not be qualified in both strains. This indicated a direct conversion of gluconeogenetic glucose-6-phosphate into glucose-1-phosphate for the formation of the cell envelope and other polymeric sugars for storage purposes. As was the case for other gluconeogenetic enzymes, the respective glucose phosphate mutase (EC 5.4.2.2) was shown to be downregulated in the mutant strain.

Internal sugar storage (*e*.*g*. glycogen formation) was not active in the mutant strain. All available glucose directly entered the glycolytic pathway as indicated by the downregulation of all enzymes necessary for polymeric sugar degradation. The downregulation of glucose phosphate mutase (EC 5.4.2.2) supported this hypothesis, since its activity is a prerequisite for transforming sugars into storage polysaccharides.

The ketoacids of the TCA serve as starter molecules for the biosynthesis of amino acids and other molecules. The removal of these ketoacids from the TCA by anabolic reaction decreases the flux of the catabolic reactions and thereby of the energetic yield. Consequently, many of the anabolic reactions starting at TCA were downregulated in the mutant strain, such as succinate dehydrogenase (EC 1.2.1.16), 4-aminobutanoate-transaminase (EC 2.6.1.19) and malate dehydrogenase (EC 1.1.1.38).

Amino acid biosynthesis pathways were qualified, but no differences between the strains were observed. The proteome data revealed that LF580 used the alpha-aminoadipate pathway for lysine biosynthesis.

#### Transport

An equivalent number of proteins linked to transport processes was up- and downregulated. Upregulated proteins were involved in the transport of amino acids as well as of oxygen into the cells. In particular, a protein (phosphate-repressible phosphate permease, g10374) responsible for the regulated uptake of phosphor was downregulated; instead a non-regulated phosphor transport is present. The regulation of intake of nitrogen (*i*.*e*. nitrate) was downregulated (nitrate assimilation regulatory protein nira, g12583) and is in accordance to the downregulation of all pathways for additional gain of nitrogen in the mutant strain.

Proteins involved in intracellular transport were downregulated in the mutant strain, for example proteins belonging to the Golgi apparatus, the peroxisome and vesicle transport.

#### Replication, morphology, cell integrity and maintenance

Growth differences were clearly reflected in the categories replication and pellet formation. As compared to the WT strain three times more proteins belonging to these two categories were upregulated in the mutant strain (Fig 3). Some proteins were downregulated, such as extracellular serine-rich protein (g7517), which was affiliated to late stage development [32]. However, changes of proteins involved in pellet morphogenesis were not observed.

The mutant strain showed some upregulated proteins involved in maintenance functions, *e*.*g*. conversion of glycine to aminolevulinate (5-aminolevulinate synthase, EC 2.3.1.37) for subsequent porphyrine synthesis. The upregulation of this enzyme might be related to enhanced respiratory chain maintenance. Higher levels of respiration in the mutant strain implicate higher levels of protection against oxidative stress. For instance, the nuclear protein qri2 nse4 (g3175), which acts in DNA repair, was upregulated in the mutant strain [33].

However, genes involved in stress response showed higher expression levels in the WT. Considering the limitation situation for the WT due to pellet formation (*i*.*e*. nutrient stress, low pH at the end of the growth phase), such stress answers would be expected [34, 35]. One example is a higher level of peroxisomal catalase (g8585), which is downregulated in the mutant strain. Further proteins in repair and stress response were downregulated: *e*.*g*. Tam domain methyltransferase (g15885) preserving the integrity of polypeptides facing age-dependent non-enzymatic reactions [36]; sphingomyelin phosphodiesterase (g8757) [37] and survival protein SurE-like phosphatase/nucleotidase (g13733). The latter may be involved in stress response, as cells with mutations in the surE gene poorly survive in the stationary phase [38].

#### Secondary metabolism

In total, 39 biosynthetic enzyme complexes were identified in the genome of *M*. *brevicaulis*. 17 were affiliated to NRPS, 18 to PKS, two to fatty acid ligase and one represented a PKS/NRPS hybrid [7, 24]. Out of these genes, 9 NRPS and 7 PKS proteins were qualified in the GO dataset. iTRAQ quantification showed a higher expression of NRPS13 and PKS15 and a lower expression of PKS 9, 14 and 18 in the mutant. However, the function of these enzymes remains unclear as the secondary metabolite profiles of WT and mutant are highly similar.

Specifically, the regulation of the respective biosynthesis pathways for the five amino acids forming scopularides A and B were compared: The pathways for l-valine, l-phenylalanine, l-alanine, d-leucine and glycine synthesis were qualified, indicating that *M*. *brevicaulis* synthesised all precursor molecules for the scopularides. This is supported by previous growth studies which showed that amino acids from nutrient sources do not serve as direct precursors for scopularide production [9]. The degradation of amino acids necessary for scopularide production was significantly downregulated in the mutant strain: Alanine degradation was downregulated at the level of alanine-glyoxylate transaminase (EC 2.6.1.44) and 4-aminobutyrate-2-oxoglutarate transaminase (EC 2.6.1.19). Valine degradation was blocked at the level of methylmalonate-semialdehyde dehydrogenase (EC 1.2.1.27). Conversion of glycine to serine (glycine hydroxymethyltransferase, EC 2.1.2.1, glycine hydroxymethyltransferase. EC 2.1.2.1) and the degradation of glycine to glyoxylate (d-amino-acid oxidase, EC 1.4.3.3, alanine-glyoxylate transaminase, EC 2.6.1.44) were downregulated resulting in high levels of glycine available for scopularide synthesis. Phenylalanine synthesis was upregulated at the level of phenylacetaldehyde conversion to phenylethylamine (primary-amine oxidase, EC 1.4.3.21). For d-leucine and serine no regulation on enzyme level were observed.

The scopularide A gene cluster contains an NRPS (NRPS1), a PKS (PKS2), a CoA ligase, an acyltransferase and a transcription factor [8]. All enzymes could be quantified, but no regulation on protein level was detected.

#### Comparison with related fungal proteomes

The resulting KEGG maps of the WT strain of LF580 and its mutant strain LF580-M26 were compared to the KEGG reference maps of *Fusarium graminearum*, *Nectria haemotococca*, *Trichoderma reesei* and *Verticillium albo-atrum*. These species are members of the Hypocreomycetidae, the same phylogenetic subclass that *M*. *brevicaulis* belongs to. The central physiological pathways of *M*. *brevicaulis* on KEGG level were found to be highly similar to the related fungi. However, six enzymes were found exclusively in *M*. *brevicaulis*, namely methylglyoxal reductase (EC 1.1.1.78), 2-methylcitrate synthase (EC 2.3.3.5) and polyphosphate kinase (EC 2.7.4.1) being part of the central catabolic metabolism, as well as aspartate racemase (EC 5.1.1.13), choline oxidase (EC 1.1.3.17) and quinate/shikimate dehydrogenase (EC 1.1.1.282). There is no obvious explanation, whether these findings are species-specific or occurred due to an inconclusive affiliation of the function using the KEGG. All proteins showed high similarities to other homologues (>56% identity on protein level) and were not affected by the changed metabolism in the mutant strain; only 2-methylcitrate synthase (EC 2.3.3.5) was downregulated.

## Discussion

### Quality of the data set for proteome study

pH, biomass and secondary metabolite production were used as parameters to identify a similar metabolic situation for both strains to find equivalent sampling points. To ensure a high coverage of the entire proteome, an adapted extraction protocol was established. Mechanical cell disruption is highly recommended for protein extraction from filamentous fungi, as reviewed by Kim *et al*. [17]. Here, two types of mechanic cell disruption were compared: beads and grinding in liquid nitrogen, the latter often being used for the extraction of fungal proteins [39–41]. While grinding in liquid nitrogen ensured a better cooling of the sample, cell disruption via beads has a very low personal deviation. Extending previous studies [23, 42, 43], we were able to show that the subsequent extraction protocol has a higher impact on the total protein yield than the initial cell disruption. We demonstrated that a higher number of proteins was obtained when solubilisation of the hardly soluble protein fraction was achieved by additional steps. For the hardly soluble fraction, a washing step using sodium carbonate was applied as first step of sample preparation to avoid the formation of micelles [44]. Denaturation with urea and thiourea supported dissolving of the proteins due to chaotrope properties [45–47]. Additional homogenisation by sonication [42] or comparable strategies [48] are recommended to enhance the solubility of proteins for the subsequent peptidic digest. The use of endoproteinase Lys-C during denaturation in urea/thiourea permitted the digest of proteins with strong secondary and tertiary bonds [49].

Approximately 30% of the theoretical protein content could be quantified applying the established protocol. Furthermore, all essential enzymes of the central metabolism were qualified in the proteome analysis, ensuring a high quality dataset.

### Hypothesis on differences between wild type and its mutant

The growth experiments led to the hypothesis that the mutation did not affect the scopularide biosynthesis, but led to a dramatic change in the growth behaviour of the mutant strain. This change included a reduction of the pellet size and a higher growth rate accompanied by a decrease of the production of acids. At the end of the rapid growth phase, the pH value of the mutant cultures increased, indicating a strong production of CO_2_ by respiration rather than production of acids as terminal products. Higher respiration rates lead to higher levels of energy yield. *I*.*e*. the mutant strain showed a higher catabolic efficiency with a main flux of carbon to the stage of oxidative phosphorylation. In this case, the rates of conversion of the tricarbonic acids to energy and new cell material would be higher in the mutant strain. All other pathways would mainly feed into the central metabolism. The proteome data supported this hypothesis: Analysis of the KEGG pathway data revealed a straight flux through the TCA in the mutant strain. Even more, proteins that are not involved were downregulated. Several enzymes including key enzymes of the affected pathways gluconeogenesis, β–oxidation and nitrogen uptake were downregulated. Therefore, no additional demand of carbon and nitrogen was supposed for the mutant. In conclusion, the mutant strain LF580-M26 apparently represents a growth mutant rather than a secondary metabolism mutant. This is strongly supported by the fact that no significant regulation of enzymes related to the scopularide biosynthesis was observed.

The structure of the pellets was the most obvious phenotypic difference between the two strains. Complex relationships between morphology and productivity are known for many fungal producers [50–55]. A higher surface-to-volume ratio in smaller pellets and different peripheral hyphae structures are assumed to support nutrient uptake [56]. For hairy and compact pellet structures, even if pellet size remained identical, completely different oxygen consumption rates were shown [54]. Thus, the different pellet morphology of the wild type might be the reason for the observed limitations.

**Fig 5:**
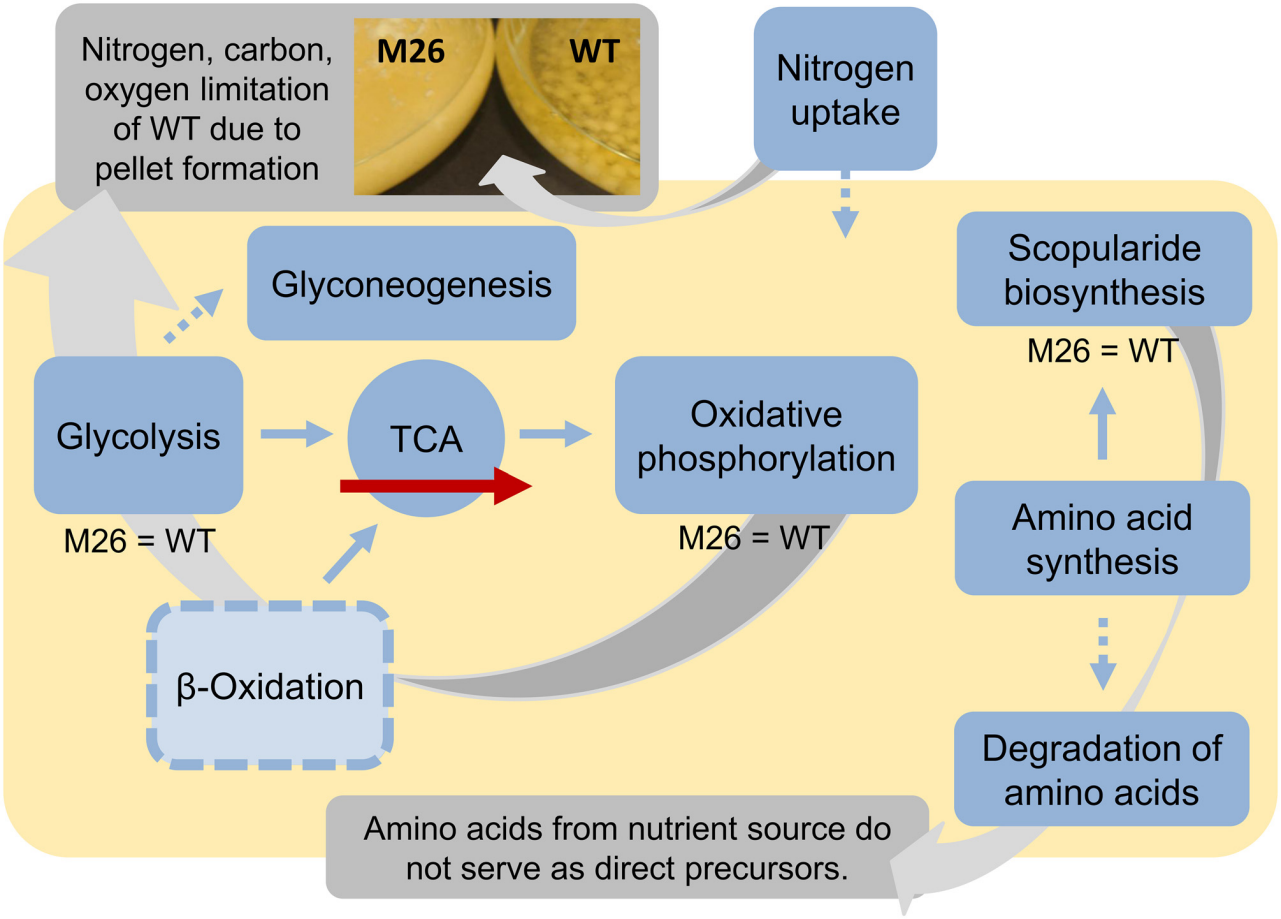
Global overview of the hypothesis of the regulatory effects in LF580-M26. Most of the KEGG pathway categories show a downregulation in LF580-M26, for example several key enzymes of the gluconeogenesis as well as isoenzymes in the TCA leading to a straighter flux. The regulation pattern in the β-oxidation is quite complex. We suggest a downregulated effect for the mutant strain. Furthermore, enzymes for additional nitrogen uptake are downregulated in LF580-M26 as well as enzymes necessary for the degradation of amino acids forming the scopularides. These observations indicate a limitation situation for the wild type strain compared to the mutant strain, which might be attributed to the change in pellet morphology.

In contrast to many other secondary metabolites [57] scopularides A and B are already produced during the rapid growth phase. The nutrient limitation was reflected in metabolite production, too: In the stationary phase, the wild type stopped production of scopularides, while the mutant strain exhibited further production. Interestingly, the application of optimised culture conditions led to a reduction of pellet size in the wild type strain [9]. The decreased pellet size resulted in a higher amount of biomass and higher production of the two scopularides. This confirms the assumption that the wild type strain was limited under the standard culture conditions and that the pellet formation is linked to this limitation (Fig 5).

Besides changes in the nutrient uptake and oxygen supply [54, 58–60], pellet formation leads to different grades of heterogeneity of the cells accumulated in the pellets [61–63]. The use of isoenzymes was seen for several pathways in the wild type strain, which might be explained by higher cell heterogeneity within the pellets. These pellets exhibit a compact structure of the peripheral region leading to an inappropriate supply of oxygen and nutrient in the inner layer [64]. A lower heterogeneity on cell level is assumed for the mutant strain due to a constant oxygen und nutrient gradient [52, 64].

Proteome data did not indicate differential regulation of proteins in pellet formation or its regulation nor allow concluding on distinct protein functions in pellet formation. It was not possible to identify specific proteins responsible for the different pellet structures of both strains. Other factors resulting from the changes might have influence on the morphological changes. The initial aggregation phase in filamentous fungi was shown to be pH-depending [52, 65]. The different pH regimes resulting from the different catabolic sets of the two strains could lead to differences in pellet formation. The regulation of the pH regimes is complex. Even in model organisms like *A*. *niger* pH regulation is not fully understood [66]. However, for *A*. *oryzae* it was shown that pellets were formed at pH values higher than 5, whereas low pH (<3.5) resulted in filamentous growth [67]. Accordingly, the mutant strain started with filamentous growth, followed by a formation of small hairy pellets after the very fast decrease of the pH of the culture.

The effects of the UV mutagenesis are quite diverse on protein level as changes were observed in the complete central metabolism of the strains. Nevertheless, the proteome analyses illustrate consequences resulting from the UV mutation. Cause and effect chains can be drawn, but hardly be traced back to a specific mutation on gene level. Like it was reviewed for the famous penicillin producer *Penicillium chrysogenum* NRRL, overproduction during an industrial strain development can originate from complex changes at proteome level [13]. Concerning LF580 and its mutant, complex changes in the central metabolism, in carbon storage, degradation of amino acids, nitrogen uptake *etc*. were found. These changes helped to explain the higher production rate of scopularides in the mutant strain.

## Conclusions

The limited number of 250 fungal proteomes (http://www.uniprot.org, August 2015) does not reflect the tremendous biodiversity of this kingdom. This study represents the first proteome analysis of a marine filamentous fungus. Proteome data of the WT strain of *M*. *brevicaulis* and its mutant strain LF580-M26 were analysed with respect to the production of the anti-cancerous scopularides A and B. Higher production levels were not caused by changes in the secondary metabolism, but could be explained by complex changes of the primary metabolism. A situation of growth limitation was present for the wild type strain under the growth conditions applied. This limitation may be caused by the structure of the pellets being the most obvious difference between the two strains. These changes could be linked to molecular changes at proteome level, directly leading to conclusions for optimisation of the biotechnological process design: For production of the scopularides, a high level of biomass production is desired, the pellet morphology needs to be controlled and growth limitation during cultivation has to be avoided. The comparative proteome analysis gave insights into the relationship of production and morphology, which are helpful for optimisation progress on strain and process level in a non-model organism.

## Materials and Methods

### Strain, cultivation and scopularide quantification

#### Strain

*M*. *brevicaulis* strain LF580 (wild type) was obtained from the strain collection of GEOMAR as cryoconserved material. The mutant strain LF580-M26 was obtained by UV radiation as described by Kramer *et al*. [10]. Ahead of the core experiment, the LF580-M26 strain was checked for its phenotypic traits by a simple cultivation test followed by an extraction and LC-MS analysis.

#### Time series for determination of equivalent sampling points

Over a period of 6 days, triplicates of cultures of LF580 and LF580-M26 (culture conditions see below) were sampled twice a day. Metabolite extraction was performed by using an equivalent amount of ethyl acetate added to culture broth and mycelium after complete homogenisation with an Ultra-Turrax (T25 basic IKA Werke, Germany) at 16000 min^-1^ for approximately 20 s. Analytical reversed-phase HPLC of extracts was carried out as described by Silber *et al*. [68]. Peak areas of scopularides A and B were automatically integrated applying the software DataAnalysis Version 3.3 (Bruker Daltonics GmbH, Germany). The biomass and the pH value of the cultures were determined by standard procedures. Pellet size was determined by visual estimation.

#### Cultivation and LC-MS of samples for the subsequent proteome analysis

Cultivation was carried out in triplicates in 300 mL Erlenmeyer flasks, containing 100 mL of modified Wickerham medium (0.3% malt extract, 0.3% yeast extract, 0.5% peptone from soy, 1% glucose × H_2_O, 3% NaCl) [69], at 28°C and 120 min^-1^ in the dark for 68 hours (WT) and 44 hours (LF580-M26), respectively. Agar pieces of 5 mm in diameter of 4-day-old (96 hours) cultures, cultivated at room temperature, were used as inoculum. Part of the culture broth was vacuum filtrated using glass microfiber filters (1.6 μm, GFA, Whatman). Remaining mycelium was transferred to a 2-mL vial, containing ceramic beads (diameter of 0.4 to 0.6 mm, Analytic Jena, Germany). 1 mL ethyl acetate was added to each sample and two 45-s homogenisation steps with a 20-s delay were performed at 6500 min^-1^ using a Precellys 24 device (Bertin Technologies, France). Afterwards, the samples were centrifuged for 10 min at 13000 min^-1^, followed by a transfer of the solvent phase to a 1.5-mL tube. Dried extracts were dissolved in methanol supported by a treatment in an ultra-sonic bath for 2 min, followed by a centrifugation step at 13000 min^-1^ for 20 s at room temperature. Samples for HPLC were taken from the upper layer. Biomass was determined after extraction of the scopularides A and B and subsequent drying at 60°C. Extract were injected (10 μL) to a Hitachi Elite LaChrom system using a C18 column (Phenomenex Onyx Monolithic C18, 100 × 3.00 mm), applying a water (A)/acetonitrile (B)/methanol (C) gradient (gradient: 0 min: 5% B, 5% C; 1.5 min: 60% B; 4 min: 90% B; 4.5 min: 100% B; flow 2 mL min^-1^) with 0.1% formic acid added to A and B. The LC system was coupled to a benchtop time-of-flight spectrometer (mircOTOF II, Bruker Daltonics). Electrospray ionisation in negative ion mode was optimised for ions in the range of *m/z* 600-750. QuantAnalysis Version 2.0 (Bruker Daltonics GmbH, Germany) was used for quantification.

### Proteome analysis

#### Harvest

Culture broth was filtrated using glass microfiber filters (1.6 μm, GFA, Whatman) to obtain mycelium, which was washed with 5 mL of ice cold 50 mM triethylammonium bicarbonate (TEAB, HPLC-grade, Sigma-Aldrich, Germany) and stored at -20°C.

#### Protein extraction

Prior to cell disruption, equal amounts of mycelium of each triplicate were combined. For liquid nitrogen cell disruption, mycelia were broken up, using a mortar and pestle until a fine powder was left. The powder was transferred to a vial containing 200 μL of 200 mM TEAB. Bead extraction was performed in 200 μL of 200 mM TEAB with a Precellys 24 device (see above); two times for 20 s at 6500 min^-1^ with a delay time of 5 s and repeated once. Supernatants of both cell disruption methods were separated by centrifugation for 10 minutes at 13000 min^-1^ at 4°C, transferred to a new vial and stored at -20°C. The remaining pellets were washed with 75 μL of 100 mM sodium carbonate three times. In between, samples were kept on ice and spun down (bead extraction) or centrifuged for 60 s at 13000 min^-1^ at 4°C (liquid nitrogen). After the final addition of sodium carbonate, samples were centrifuged for 10 minutes at 13000 min^-1^ at 4°C. All protein extraction procedures were carried out with ice cold buffers, solutions and instruments in order to inhibit undesired protease activity.

#### Sample preparation for proteome analysis

Precipitation of the supernatant fraction (“easily soluble” protein fraction) was performed using 1 volume of a mixture of methanol, chloroform and water (4:1:3) [70]. Samples were dissolved in 50 μL of 50 mM TEAB in a sonication bath for 20 min. Protein bonds were reduced and alkylated with dithiothreitol (DTT, Sigma-Aldrich, Germany) at a final concentration of 5 mM in 200 mM TEAB for 40 min and iodoacetamide (IAA, purity ≥ 99%, Sigma-Aldrich, Germany) at a final concentration of 15 mM in 200 mM TEAB for 40 min in the dark. Pellet fractions (“hardly soluble” protein fraction) were dissolved in 50 μL of 8 M urea (containing 6 M urea (Sigma-Aldrich, Germany) and 2 M thiourea (*ReagentPlus* ^®^, Sigma-Aldrich, Germany)) in 50 mM ammonium bicarbonate (*ReagentPlus* ^®^, Sigma-Aldrich, Germany), supported by a treatment in a sonication bath for 20 min. DTT and IAA were added for the same duration at a final concentration of 20 mM and 40 mM in 8 M urea. rLys-C (mass spec grade, Promega, USA) was added in a ratio of 1:30 to the sample for 4 hours at 37°C. The soluble fraction, as a combination of all supernatants yielded during the washing procedure of the initially insoluble fraction, is referred to as “easily soluble” in the manuscript. The pellet fraction (“hardly soluble” protein fraction) was diluted 1:10 before trypsination. Trypsination (sequencing grade modified trypsin, Promega, USA) was performed for both fractions with an enzyme-protein ratio of 1:30 at 37°C over night. The samples were acidified with formic acid to a final concentration of 5%.

#### Total protein analysis

Protein amount was determined using Roti^®^-Quant (Carl Roth, Germany) in 96 well microtiter plates, according to Bradford [71]. Bovine serum albumin served as standard. SDS-PAGE was carried out in 12% gels [72], followed by staining with Coomassie Brilliant Blue (0.03% in 10% acetic acid).

#### Proteome analysis

Peptide samples were isotopically labelled using iTRAQ (reporter ions *m/z* 115 and *m/z* 117), mixed and further fractionated using hydrophobic interaction liquid interaction chromatography (HILIC) on a Dionex Ultimate 3000 nano/capillary HPLC system (Thermo Scientific, USA): The samples were loaded onto a custom made HILIC column packed with TSKgel Amide-80 (3 μm bead size, 10 cm length, 300 μm ID, Tosoh Bioscience LLC, USA). The resulting 15 fractions were analysed in duplicates by nano-LC-MS/MS using a Dionex UltiMate 3000 nano-HPLC coupled to a Thermo Scientific Orbitrap Q-exact mass spectrometer (Thermo Scientific, Germany). The samples were separated using nano-HPLC as previously described [73]; the samples (5 μL) were loaded onto a custom made fused capillary pre-column (2 cm length, 360 μm OD, 75 μm ID) with a flow of 5 μL min^-1^ for 7 min. Trapped peptides were separated on a custom made fused capillary column (20 cm length, 360 μm outer diameter, 75 μm inner diameter) packed with ReproSil Pur C18 3-μm resin (Dr. Maish, Germany) with a flow of 300 nL min^-1^ using a linear gradient from 95% solution A (0.1% formic acid) to 35% B (100% acetonitrile in 0.1% formic acid) over 86 min followed by 10 min at 90% B and 14 min at 98% A at a flow rate of 300 nL min^-1^. Mass spectra were acquired in positive ion mode applying automatic data-dependent switch between one Orbitrap survey MS scan in the mass range of 300 to 1500 *m/z*, followed by HCD fragmentation and Orbitrap detection of the ten most intense ions observed in the MS scan. Target value in the Orbitrap for MS scan was 1000000 ions at a resolution of 70000 at *m/z* 200. Fragmentation in the HCD cell was performed at normalised collision energy of 27 eV for non-labelled peptides and 30 eV for iTRAQ-labelled peptides. Ion selection threshold was set to 50000-100000 counts. Selected sequenced ions were dynamically excluded for 60 s. Samples were analysed in duplicates.

#### Data analysis

A combined MASCOT-SEQUEST search was performed. Peak lists (mgf files) were processed using the Proteome Discoverer 1.4, version 1.4.0.288 with following search parameters: MS accuracy 10 ppm, MS/MS accuracy 0.1 Da for HCD data with two missed cleavages allowed, fixed modification of cysteine blocked with carbamidomethyl, and—in case of iTRAQ-labelled specimens, lysine and N-terminal iTRAQ—and variable modifications: methionine oxidation and deamidated asparagine and glutamine. Tandem mass spectra were searched against the annotated genome sequence of strain LF580 [7, 24]. False discovery rates were obtained using percolator selecting identification with a q-value equal or less than 0.01. iTRAQ quantification was performed using Proteome Discoverer with reporter ion area integration in a 20 ppm window. Ratios were normalised against the median peptide ratio. Peptide sequences were scanned using BLASTP [74] at E-value <1e^-3^ in the Blast2GO framework [25]. Annotation analysis was performed by a coupled GO and KEGG pathway analyses [26] of upregulated and downregulated proteins by homology detection scan against the *M*. *brevicaulis* LF580 genome [7, 24] using the Blast2GO tool [25].

## Supporting Information

S1 File Table of analysed proteins from *M*. *brevicaulis* LF580 and its mutant LF580-M26. Qualification and quantification of proteins of LF580 were determined in a MS-MS based proteome analysis (Table A). An iTRAQ labelling approach was used to compare the proteomes of LF580 and LF580-M26 (Table B). Proteins were associated to functionality using a Gene ontology analysis (Table C) and a KEGG pathway analysis (Table D).
(XLSX)
